# Acquired Resistance to Immune Checkpoint Blockades: The Underlying Mechanisms and Potential Strategies

**DOI:** 10.3389/fimmu.2021.693609

**Published:** 2021-06-14

**Authors:** Binghan Zhou, Yuan Gao, Peng Zhang, Qian Chu

**Affiliations:** Department of Oncology, Tongji Hospital, Tongji Medical College, Huazhong University of Science and Technology, Wuhan, China

**Keywords:** immune checkpoint blockade therapy, acquired resistance, neoantigen depletion, interferon signaling aberration, tumor-induced exclusion, immunosuppression, tumor cell plasticity, treatment modalities.

## Abstract

The immune checkpoint blockade therapy has completely transformed cancer treatment modalities because of its unprecedented and durable clinical responses in various cancers. With the increasing use of immune checkpoint blockades in clinical practice, a large number of patients develop acquired resistance. However, the knowledge about acquired resistance to immune checkpoint blockades is limited and poorly summarized. In this review, we clarify the principal elements of acquired resistance to immune checkpoint blockades. The definition of acquired resistance is heterogeneous among groups or societies, but the expert consensus of The Society for Immunotherapy of Cancer can be referred. Oligo-progression is the main pattern of acquired resistance. Acquired resistance can be derived from the selection of resistant cancer cell clones that exist in the tumor mass before therapeutic intervention or gradual acquisition in the sensitive cancer cells. Specifically, tumor intrinsic mechanisms include neoantigen depletion, defects in antigen presentation machinery, aberrations of interferon signaling, tumor-induced exclusion/immunosuppression, and tumor cell plasticity. Tumor extrinsic mechanisms include upregulation of other immune checkpoints. Presently, a set of treatment modalities is applied to patients with similar clinical characteristics or resistance mechanisms for overcoming acquired resistance, and hence, further research is required.

## Introduction

Since the approval of the first CTLA-4 blockade by the US Food and Drug Administration (FDA) in 2011 (1, 2), immune checkpoint blockades (ICBs) have completely changed cancer treatment modalities (3–6). Due to more extensive use of ICBs in clinical practice, several patients exhibit disease progression, although with an initial response, termed as acquired resistance (AR). However, the underlying mechanisms of AR and strategies for overcoming AR are limited and poorly summarized. Previous research (7–9) has focused on primary resistance to ICBs, providing limited description about AR, whose mechanisms and countermeasures differ from those of primary resistance. In addition, updated information on AR needs to be emphasized.

In this review, we clarify the principal elements of AR to ICBs including the (1) definition of AR; (2) clinical characteristics of AR; (3) underlying mechanisms of AR; and (4) potential strategies to overcome AR. We hope that this review will help researchers and clinicians recognize AR to ICBs more comprehensively and in detail.

## Definition of AR to Immune Checkpoint Blockades

Primary resistance occurs when cancer cells do not respond to immunotherapy, whereas AR normally occurs as disease progression after an initial response. Contrary to primary resistance, the current definition of AR is highly heterogeneous among groups or societies. Different researchers define AR differently when they explore the underlying mechanisms or design clinical trials, with the main controversies being prior response (10, 11) (whether the stable disease should be regarded as a response to ICBs), treatment duration time (10, 12) (a cut-off time to distinguish from primary resistance), and treatment discontinuation (10, 13) (whether disease progression after discontinuation to ICBs should be regarded as AR). These divergent views have obstructed the integrated interpretation of each solitary study and further research in this field. To clarify the underlying resistance mechanisms to immunotherapy, Sharma et al. introduced a three-category classification of primary resistance, adaptive immune resistance, and AR (7). However, adaptive immune resistance can manifest like primary resistance or AR in the clinic, and they did not clearly define these terms (7). The International Association for the Study of Lung Cancer (IASLC) expert panel has defined primary resistance (no objective tumor radiographic response and treatment duration < 6 months) and AR (an objective tumor response and treatment duration ≥ 6 months) for lung malignancies immunotherapy, but they did not consider other clinical settings in addition to advanced disease or the issue of treatment discontinuation (14). The Society for Immunotherapy of Cancer (SITC) proposed expert consensus definitions for PD-(L)1 inhibitor monotherapy resistance in solid tumors (15) (**Table 1**), which can be widely applicable. However, definitions based on empirical data from clinical trials, patient registries, or previous studies investigating treatment beyond progression and/or treatment after relapse are still needed, which will fuel future research on AR to ICBs, providing guidelines for clinical practice.

**Table 1:** The definitions for PD-(L)1 inhibitor monotherapy resistance in solid tumors^a^.

Definitions of neoadjuvant therapy resistance		
	Major pathological response^b^	Other requirements
Primary resistance	No	Follow primary resistance definitions
Acquired resistance	Yes	Follow acquired resistance definitions
**Definitions of adjuvant therapy resistance**		
	Timing of last dose prior to PD	Confirmatory biopsy^c^
Primary resistance/early relapse	<12 weeks	Yes
Late relapse	≥12 Weeks	Yes
**Definitions of primary and acquired resistance in advanced disease setting**		
	Drug exposure and best response	Confirmatory scan
Primary resistance	≥6 weeks; PD, SD for <6 months^d^	Yes, at least 4 weeks after progression^e^
Acquired resistance	≥6 months; CR, PR, SD for >6 months^d^	Yes, at least 4 weeks after progression^e^
**Definitions of resistance after discontinuing treatment for metastatic disease** ^f^		
	Best response and duration of time after last dose of PD-(L)1 inhibitor	Confirmatory scan
Primary resistance	No CR/PR prior to discontinuation	No
Acquired resistance	Prior CR/PR and ≤12 weeks from last dose	Yes
Late progression	Prior CR/PR and >12 weeks from last dose	Yes

a Adapted from SITC expert consensus for PD-(L)1 inhibitor monotherapy resistance in solid tumors (15).

b Tumor shrinkage ≥90%.

c In this setting, a confirmatory biopsy would supplant a confirmatory scan.

d Indolent tumor types might require modification of the timeframe.

e Other than when tumor growth is very rapid and patients are deteriorating clinically.

f The reason for treatment discontinuation may be CR, PR, end of study, or other social rationales.

CR, complete response; PD, progressive disease; PR, partial response; SD, stable disease. Per Response Evaluation Criteria in Solid Tumors 1.1.

AR is considerably different from primary resistance to ICB therapy based on three aspects— (1) immunophenotypes: AR mainly occurs in inflamed tumors, while primary resistance occurs in excluded or desert tumors, which lack sufficient immune infiltration (16); (2) underlying mechanisms: AR is attributed to the mutual evolution of tumor and tumor microenvironment (TME) under the pressure of ICB-activated immune elimination, while primary resistance can be attributed to the host, tumor, TME, and microbiome, a reflection of baseline status resisting ICBs (17–19); and (3) coping strategies: the coping strategies of AR could be specific to the resistance mechanisms, aiming at extending the patients’ survival, while the coping strategies of primary resistance are comprehensive and largely dependent on heating the cold tumor (20).

## Clinical Characteristics of AR

Unlike primary resistance, the rates of AR have not been routinely reported and thus are not characterized across tumor types. Based on available data of response duration time, Schoenfeld et al. inferred the AR rate and found that AR rate ranges from 11% to 71% among different tumor types, with the median of 39%, and a tumor with a lower response rate has a higher AR rate (21). Additionally, based on clinical trial data, AR to nivolumab in melanoma at 5-year follow-up was 39% (22), while AR to nivolumab in non-small-cell lung cancer (NSCLC) at 4-year follow-up was 65% (23).

In the population of patients with response to ICBs, the median duration of response (DOR) was heterogenous among different tumor types, lines of therapies, or PD-L1 levels. Patients with melanoma had the longest DOR, with the median of more than four years. While the median DOR in other tumor types was usually less than two years, indicating that more than half responders would progress within two years (**Supplementary Table 1**).

In the population of patients with AR to ICBs, the clinical characteristics of these patients were not routinely reported in clinical trials. Based on the limited clinical trials and real-world studies on this topic (24–28), AR is more likely evident in patients with prior partial response (PR) instead of complete response (CR) (stable disease is not taken into consideration). The median time to resistance is inconsistent and needs further validation. Oligo-progression is the main pattern of resistance. The median survival time after AR seems heterogeneous among different patients and largely depends on the successive treatment (**Table 2**). However, it must be noted that the definition of AR in these studies is either not given or different from the SITC recommendations, without the requirement of 6-month drug exposure and 3-month limit for drug discontinuation.

**Table 2:** Clinical characteristics of acquired resistance to ICBs.

Study	Cancer type	No. of patients	Treatment	Prior response	Median time to resistance	Pattern of progression	Median post-progression survival
Wang et al. (24)	Melanoma	36	Anti-PD-1 monotherapy, any line	11% CR, 89% PR	11.1m	78% single site	12.8m
Pires da Silva et al. (25)	Melanoma	12	Anti-PD-1 plus anti-CTLA-4, first line	50% PR, 33% SD, 17%, pseudo-progression	9.6m	Median of 5 progressing lesions	Not reach, One-year survival rate is 83%
Keynote 006^*^ (26)	Melanoma	27	Anti-PD-1 monotherapy, anti-CTLA-4 monotherapy, first or second line	22% CR, 59% PR, 19% SD	33.3m	60% single site, 20% double sites	NA
Gettinger et al. (27)	NSCLC	26	Anti-PD-(L)1, anti–PD(L)1 plus anti-CTLA4, anti–PD-1 plus erlotinib, any line	100% PR	10.4m	54% single site, 35% double sites	Not reach, three-year survival rate is 70%
Shah et al. (28)	NSCLC	33	Anti-PD-1 monotherapy, any line	NA	NA	67% single site	NA

^*^26 patients completed two-year treatment of pembrolizumab, and 1 patient did not complete two-year treatment of pembrolizumab for achieving complete response. The time to resistance was calculated from the end of pembrolizumab treatment. The pattern of progression of two patients were not available, so they are excluded to calculate the rate of oligo-progression. NA, not available.

## Underlying Mechanisms of AR

**Figure 1:**
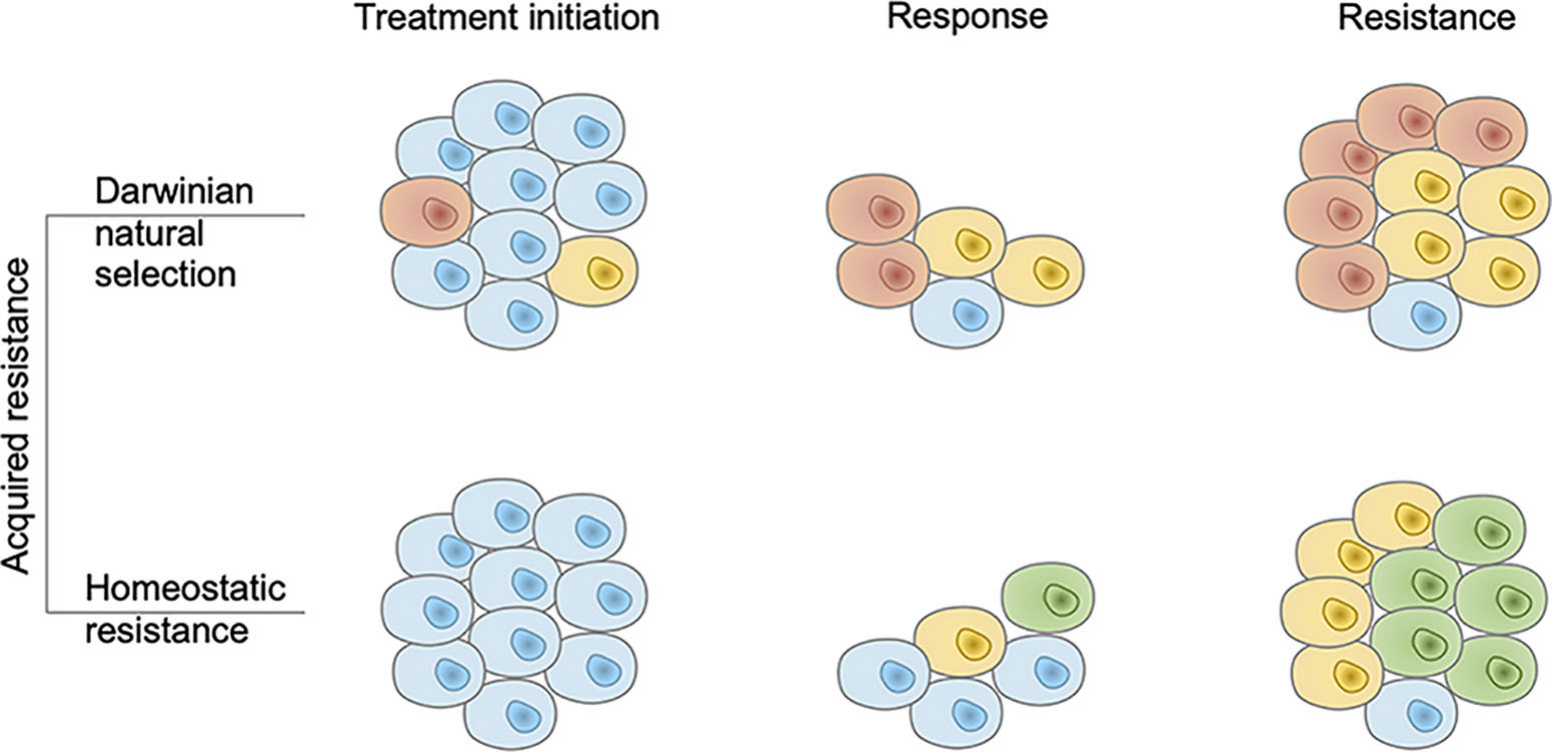
Two modes of acquired resistance to immunotherapy. In the Darwinian natural selection mode, immunotherapy resistant tumor cell clones pre-exist in the tumor mass. They are present at the treatment initiation phase and resist the immune response. In the homeostatic resistance mode, resistant clones are not present before the treatment initiation but emerge under the additional immune pressure it generates. Brown, yellow, and green cells denote different resistant tumor cell clones and blue cells denote sensitive tumor cell clones.

Based on the origin of AR, AR to immunotherapy can be divided into two major categories, though the phenotype of these two categories is almost similar in the response and resistance phases (**Figure 1**). The first type of resistance is a special form of Darwinian natural selection that comes from the selection of genetic or epigenetic heritable traits that exist in the tumor mass before a therapeutic intervention (29). Intratumor heterogeneity can come from numerical or structural chromosomal instability, somatic mutagenesis, and epigenetic diversity (30). AR-related heterogeneity lies in various aspects of anti-tumor immunity. Additionally, the quantity and quality of resistant tumor cell clones reflect the forms of resistance. The abundant and high-quality resistant clones provide a basis for the occurrence of primary resistance, while scarce and low-quality resistant clones retain the possibility for AR.

PD-L1 expression is broadly used as a biomarker for the initiation of PD-(L)1 inhibitor therapy, approved by the US FDA, although it has many shortcomings (17). However, PD-L1 expression is associated with T cell infiltration and forms a scattered but not disseminated pattern in tumor tissues (31), which can result in different responses of the individual tumor cell to PD-(L)1 inhibitor. PD-L1 expression also shows heterogeneity among different anatomic sites and decreases after ICB therapy (32). Tumor cells can express neoantigens, resulting from the instability of the genome, which can be recognized by the cytotoxic immune cells and elicit effective anti-tumor immunity (33–35). Similarly, neoantigens exhibit intratumor heterogeneity, and the clonal neoantigen load rather than the subclonal one can predict ICB-treated patient survival (36, 37). Single-cell RNA-Seq data from lung adenocarcinoma patients and cell lines revealed the intratumor heterogeneity of IFN-γ signaling pathway genes and tumor antigen expression levels (38). Proteomic analysis of the single cell-derived tumor organoids also revealed that the human leukocyte antigen (HLA) class I peptides are heterogeneous within a tumor mass (39). Rosenthal et al. reported 64 untreated early-stage NSCLC patients in the TRACERx 100 cohort with 164 tumor regions and found a great heterogeneity of the immune infiltration status among different regions of the same tumor in the same patient (40). The different immune infiltration status also leads to different immune editing levels (40).

After the initiation of ICBs, responders experienced genomic contraction, loss of the tumor cells expressing specific neoantigens, expansion of specific T cells targeting the corresponding neoantigens, and upregulation of other checkpoints (such as TIGIT, TIM-3, and VISTA), with the left cancer clones more resistant to the activated immunity (41). Gu et al. established a unique mouse system that utilized clonal tracing and mathematical modeling to monitor the growth of each cancer clone in response to ICBs, revealing that ICB-resistant cancer clones pre-exist in the tumor mass and finally result in AR (42). The smaller the tumor size, the more abundant are the ICB-resistant clones (42). One of the identified resistant clones had higher expression of genes involved in DNA replication and sterol biosynthesis and lower expression of those involved in ribosome biogenesis. The other resistant clone had a higher expression of genes involved in interferon response and lower expression of those involved in growth factor binding. Both clones had higher expression of genes indicating cytotoxic T cell dysfunction, while the MHC-I expression and the sensitivity to IFN-γ were normal (42). Additionally, Darwinian natural selection can be exemplified by the dissociated responses (43) to ICBs and the phenomenon that patients with prior PR are more likely to acquire resistance to immunotherapy compared to those with CR (**Table 2**).

The second type of resistance to immunotherapy is AR in sensitive tumor cells, also called “homeostatic resistance” (29). It is easy to understand that adaptive changes can occur within tumor cells to help them survive the drug-induced massacre. In the field of target therapy for lung cancer, epidermal growth factor receptor (EGFR) T790M mutation gradually emerges with the initial mutation of 19del or L858R unchanged, thus leading to AR to the first- or second-generation tyrosine kinase inhibitors (TKIs) (44, 45). One example of this type of resistance for immunotherapy is that tumor cells can upregulate PD-L1 under the pressure of the immune cell-secreted IFN-γ (46).

**Figure 2:**
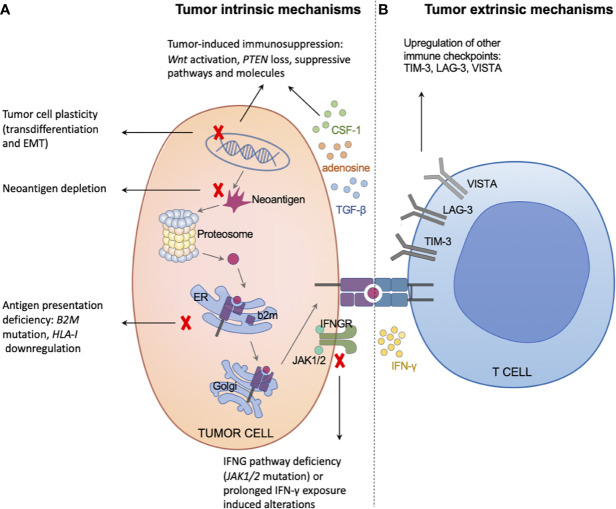
Tumor intrinsic and extrinsic mechanisms of acquired resistance to immunotherapy. **(A)** The left panel illustrates tumor intrinsic mechanisms of acquired resistance, including neoantigen depletion, defects in antigen presentation machinery, interferon signaling deficiency or prolonged exposure, tumor-induced exclusion/immunosuppression, and tumor cell plasticity. **(B)** The right panel illustrates tumor extrinsic mechanisms of acquired resistance, mainly through upregulating other immune checkpoints, such as TIM-3, LAG-3, and VISTA.

It is difficult to study the underlying mechanisms of AR following the two categories mentioned above, although they are easy to understand. Regarding a specific mechanism underlying AR (such as a certain gene mutation), researchers have not been able to determine whether this trait pre-exists in the tumor or is truly acquired at the individual cell level (high-resolution technologies will fuel the related research, such as single-cell RNA-seq (47) and spatial multi-omics (48)). It seems that Darwinian natural selection has a greater contribution to AR than homeostatic resistance (49, 50); however, the question remains unanswered. Furthermore, the two categories mentioned above do not completely fulfill the role of immune cells, which is fundamental for anti-tumor immunity. Thus, we classified the underlying mechanisms of AR to immunotherapy into tumor intrinsic factors and extrinsic factors (**Figure 2**).

### Tumor Intrinsic Mechanisms Underlying AR

#### Neoantigen Depletion

High-quality neoantigens are crucial for the efficacy of ICBs (37), and their depletion results in AR. Under the pressure of immune elimination, cancer cells can escape immune attack through the HLA loss of heterozygosity (LOH) or neoantigen depletion (40, 51). The neoantigen depletion is more pronounced in patients with intense immune infiltration or with *HLA* intact than *HLA* LOH (40). In a study (10) including four NSCLC patients with AR to ICBs, the comparison between pre-treatment and post-treatment samples revealed that some predicted neoantigens were lost in the post-treatment samples with no significant differences in the expression of PD-L1, PD-1, CTLA-4, or genetic aberrations related to immune responses. In addition, the lost neoantigens have high affinity to MHC and TCR, which can stimulate the T cells *in vitro* in the corresponding patients (10). Similarly, another group (52) also identified the reduced expression of high-immunoreactivity neoantigens in a metastatic uterine leiomyosarcoma patient with AR to PD-1 blockade, although the biallelic PTEN loss was also detected in the treatment-resistant tumor, indicating that the same tumor can resist immunotherapy through more than one mechanism. Notably, tumor antigen depletion is also a usual resistance mechanism in T cell transfer therapy (53, 54). However, it remains elusive whether the depletion of neoantigens is a consequence of Darwinian natural selection with the neoantigen-expressing clones eradicated or is a biological adaptation at an individual cancer cell level; the former seems inevitable for cancer immunotherapy, the strategies of which are inducing new immunogenicity, while the latter can be overcome by the modulation of certain molecular events.

#### Defects in Antigen Presentation Machinery

Tumor antigens can only be recognized by the cytotoxic T lymphocyte after combining with MHC-I and being presented on the cancer cell surface (55), while the defects in this process result in AR. The effective antigen presentation machinery depends on the normal functions of HLA-I, TAP, beta-2-microglobulin (B2M), and other molecules (56). Under the pressure of immune infiltration, cancer cells can evade immune attacks through aberrations of antigen-presenting genes; furthermore, the mutations of *HLA* genes are more likely to occur in the TCR-binding domain (57, 58). In a study (12) including four metastatic melanoma patients with AR to pembrolizumab, homozygous *B2M* frame-shift deletion was detected in a resistant tumor sample, with IHC validating the loss of MHC class I heavy chain at the cancer cell outer-membrane, even though diffuse intracellular staining indicated continued production of MHC class I molecules, in line with the transporting and stabilizing function of beta-2-microglobulin. *B2M* and the location of MHC-I were normal in the pre-treatment sample of this patient (12). The biallelic loss of *B2M* (homozygous mutation or heterozygous mutation combined with LOH) in the resistant tumor is also confirmed by other groups (13, 59).

However, the efficacy of ICBs is heterogeneous among patients with *B2M* LOH, for retaining a wide type allele of *B2M.* In a study by Sade-Feldman et al. (59), *B2M* LOH was detected in both the pre-treatment tumor and the post-treatment tumor of Pat 99, while the beta-2-microglobulin protein was lost only in the post-treatment sample, in line with the patient acquiring resistance after a prior response. In Pat 25, *B2M* LOH and beta-2-microglobulin protein loss were both detected in the pre-treatment tumor and post-treatment tumor, with the patient primarily resisting therapy (59). In a larger cohort, *B2M* LOH was also detected in patients with response to ICBs (4/36, 11.1%), although it mainly occurred in patients resistant to ICBs (20/69, 28.9%), with a significant difference (p = 0.03) (59). Additionally, *B2M* LOH can predict a worse survival to checkpoint blockade therapy (59). These phenomena indicate that *B2M* LOH can increase the possibility of beta-2-microglobulin loss, and the protein loss can result in resistance to ICBs, while *B2M* LOH solely cannot. The retained wide type allele of *B2M* may undergo epigenetic modification. Similarly, the relationship between single allelic truncating *B2M* mutation and AR remains elusive.

In contrast, some studies reported the inessential role of *B2M* in ICBs immunotherapy. Three retrospective studies of patients with colorectal carcinoma both found that a *B2M* mutation was more likely to happen in the microsatellite instability-high (MSI-H) patients and the *B2M* status was not associated with tumor immune infiltration (60–62). Furthermore, Middha et al. reported that *B2M* status was not associated with MHC-I expression and the MSI-H patients with inactivating mutation of *B2M* can still respond to ICBs, with the beta-2-microglobulin loss validated by IHC (62). Based on a study of patients with mismatch repair deficiency (d-MMR) and its supplementary information (11), the mutation of *B2M* can also be detected in patients with response to ICBs, though the IHC results were not available. The probable explanation of these phenomena is that the neoantigen-rich tumors (MSI-H or d-MMR) can present the antigen, independent of beta-2-microglobulin or beta-2-microglobulin, playing a different role in primary resistance compared to AR. Rizvi et al. also reported a patient with biallelic *B2M* mutation responding to anti-PD-1 therapy, with IHC validating the beta-2-microglobulin loss (63). However, detailed information was not available to evaluate the status of MSI or MMR (63). The relationship between beta-2-microglobulin, MHC-I, and cancer immune response needs further exploration, especially in the MSI-H or d-MMR tumors.

HLA-I is another component of antigen presentation machinery. There are limited reports about the contribution of *HLA-I* mutation to the efficacy of ICBs for the high polymorphism of the *HLA* loci (64), but it seems that there are still some recurrent mutations that are positively selected to resist immune attack (57, 64). The two studies (65, 66) contradicted each other about whether LOH of *HLA-I* is associated with response or resistance to ICBs, though it is widely accepted that *HLA* LOH is a strategy of cancer cells to evade immune attack (51) and the *HLA*-corrected TMB shows better predictive value than TMB (67). In a case of T cell transfer therapy targeting mutant *KRAS* (68), the patient progressed after the prior response to the injected HLA-C*08:02–restricted tumor-infiltrating lymphocytes. The resistant lesion was resected and found to have lost the chromosome 6 haplotype encoding the HLA-C*08:02 class I major histocompatibility complex (MHC) molecule (68). However, high-grade evidence is still needed to illustrate whether *HLA-I* LOH can result in AR to ICBs. Some studies reported the relationship between transcriptional downregulation of MHC class I molecule and resistance to immunotherapy. Paulson et al. (69) reported two patients with AR to combined immunotherapy (T cell transfer combined with ICBs). Comparison of the pre-treatment and post-treatment tumor from the first patient revealed the transcriptional loss of *HLA-B* with the injection of *HLA-B* restricted CD8+ T cells (69). The resistant tumor from the second patient transcriptionally downregulated HLA-A with the injection of HLA-A restricted CD8+ T cells (69). Furthermore, the transcriptional loss of *HLA-I* can be rescued by hypomethylating agents (69). Lee et al. (70) reported that MHC class I downregulation was a hallmark of resistance to PD-1 inhibitors and was associated with the MITF^low^/AXL^high^ de-differentiated phenotype and cancer-associated fibroblast signatures. In addition, the resistant phenotype was driven by TGB-β (70). Mechanically, the transcriptional loss of *HLA-I* can be mediated by PRC2 (71). It is worth noting that some emerging factors can also affect the antigen presentation machinery, such as HPV16 E5 (72), IL-8 (73), and autophagy (74). The relationship between these factors and AR needs to be researched in the future.

#### Dual Effects of Interferon Signaling

IFN-γ plays a pivotal role in ICB therapy, including directly killing cancer cells (75), upregulating MHC-I (76), upregulating PD-L1 (46), and other immune-modulating functions (77). The intact IFN-γ pathway includes *IFNGR1*, *IFNGR2*, *JAK1*, *JAK2*, *STAT1*, and the downstream response elements, as well as negative modulating elements such as *SOCS-1* (77, 78). Gao et al. found that melanoma tumors resistant to CTLA-4 inhibitor contain genomic defects in IFN-γ signaling genes and confirmed that the knockdown of the *IFNGR1* gene in B16/BL6 tumors diminished the efficacy to CTLA-4 inhibitor (79). Similarly, the biallelic *JAK1/2* loss-of-function mutation leads to the defects of IFN-γ induced PD-L1 expression, resulting in primary resistance to PD-1 blockades (80). In the study of four melanoma patients with AR to pembrolizumab (12), the resistant tumors of two patients were found to harbor *JAK1 or JAK2* truncating mutation combined with LOH, leading to the biallelic loss of function, while no *JAK1/2* mutations were seen in the baseline tumors. On the functional level, the *JAK1/2* loss of function leads to the defects of IFN-γ induced growth arrest, MHC-I expression, and PD-L1 expression (12). The findings were also validated by another group (13). Moreover, the genomic analysis revealed that the loss of tumor suppressor *CDKN2A* can enhance the susceptibility of *JAK2* loss, increasing the rate of AR (81). However, Luo et al. reported the difference between *JAK1* and *JAK2*, with *JAK1* deficiency being able to mediate resistance to anti-PD-L1 immunotherapy while *JAK2* could not (82), consistent with a recent report that metastases with *JAK2* loss-of-function might be regressing under immunotherapy (83). As for the LOH or heterozygous mutations of the IFN-γ signaling genes, retaining a wide type allele, the tumors are still under effective immune attack (84, 85).

Although the defects of IFN-γ signaling can drive AR, the prolonged interferon signaling can also elicit resistance to ICBs through multiple inhibitory pathways, such as upregulation of IDO and other immune checkpoint ligands (86). IFN-γ serves as an essential element for cytotoxic T cell-dependent cancer genome immunoediting (87). In the preclinical study (88), IFN-β can increase the tumor infiltration of regulatory T cells *via* NOS2, resulting in immunosuppression and AR. Besides, IFN-β can coordinate with all-trans retinoic acid to upregulate CD38, resulting in resistance *via* the adenosine receptor signaling (89).

#### Tumor-Induced Exclusion/Immunosuppression

The immune desert tumors are well-known to resist immunotherapy due to the lack of pre-existing immune elements (90). Spranger et al. first reported that the tumor-intrinsic *Wnt/β-catenin* activation is the main cause of immune desert (91). Besides, Peng et al. reported that loss of *PTEN* can decrease the immune infiltration, thus promoting the formation of immune desert (92). *PTEN* loss of function occurs *via* the PI3K-Akt pathway (93). In the study of Peng et al. (92), *PTEN* loss resulted in primary resistance in mice receiving adoptive cell therapy, and *PTEN* loss positively correlated with primary resistance in a patient cohort receiving anti-PD-1 therapy. As for AR, George et al. reported a patient with metastatic uterine leiomyosarcoma receiving anti-PD-1 monotherapy, the resistant sample of which was detected with biallelic *PTEN* loss (52). Trujillo et al. also reported biallelic *PTEN* loss as an AR mechanism for a patient with melanoma receiving combined immunotherapy of anti-PD-1 and anti-CTLA-4 (94). They also reported a patient with melanoma receiving melanoma-peptide/interleukin-12 vaccine, the resistant sample of which was detected with *β-catenin* activation (94). In the cohort of melanoma patients with AR to ICBs (95), *PTEN* loss was detected in four patients and *β-catenin* activation was detected in two patients, without overlap.

In addition to genetic changes, tumor cells can also induce immunosuppression to resist immunotherapy *via* multiple approaches. Under the pressure of ICBs-induced immune activation, tumor cells can increase the infiltration of regulatory T cells *via* IFN-β/NOS2 (88), recruit myeloid-derived suppressor cells (MDSCs) *via* PD-L1-NLRP3-Wnt5a-CXCR2 (96), and secrete CSF-1 to increase the level of tumor-associated macrophages (TAMs) (97). However, it remains elusive whether CSF-1 induced TAMs are immunoreactive or immunosuppressive (16). The clinical trial of anti-CSF-1R antibody largely failed (98). Intriguingly, TAMs can also steal anti-PD-1 mAbs to alleviate efficacy (99). Kim et al. identified two immune subtypes of triple-negative breast cancer, including neutrophil-enriched (NES) and macrophage-enriched subtypes (MES), and the MES-to-NES conversion can mediate acquired ICBs resistance (100). Furthermore, tumor-derived bone morphogenetic protein 7 (BMP7), a member of the TGF-β superfamily, promotes resistance to immunotherapy (101). Adenosine is considered an important immunosuppressive cytokine (102). During the treatment of PD-1 inhibitors, tumor cells can upregulate CD73 to increase adenosine production, leading to AR (103). Moreover, the upregulation of CD73 was validated in the resistant tumor from a melanoma patient with initial CR to pembrolizumab, while CD73 was at a relatively low level in the baseline tumor (103). Similarly, tumor cells can also upregulate CD38 to promote adenosine production, leading to AR (89). Indoleamine 2,3 dioxygenase (IDO) is the rate-limiting enzyme that catabolizes tryptophan (Trp) into kynurenine (Kyn), controlling the function of T cells (104). The upregulation of IDO in tumor cells can also induce immunosuppression, resulting in resistance (105, 106).

#### Tumor Cell Plasticity Drives Acquired Resistance

Tumor cell plasticity represents the ability of tumor cells to undergo phenotypic changes in response to environmental stimuli without modifying their genome. The contribution of tumor cell plasticity to the acquired anti-tumor therapy resistance has been recognized recently (107, 108).

One important issue is transdifferentiation. The transdifferentiation of non-small-cell lung cancer (NSCLC) to small-cell lung cancer (SCLC) is considered to be one of the AR mechanisms to target therapy (109). It is thought that this transdifferentiation depends on the inactivation of *RB1*, without the dependency of *EGFR* status or tyrosine kinase inhibitors use (109). There are indeed several case reports about the ICBs induced transdifferentiation of NSCLC to SCLC (110–112). There were five patients in total. Four of them were treated with nivolumab and one with pembrolizumab. After progression on ICB, three of them received EC chemotherapy (etoposide-carboplatin) and all responded. However, the ICBs induced transdifferentiation and the underlying mechanisms data from large cohorts is yet to be confirmed.

Another issue is the epithelial−mesenchymal transition (EMT). EMT is thought to be associated with extensive anti-tumor therapy resistance (107, 108, 113). Sehgal et al. found that immunotherapy persister cells, which can resist CD8 T-cell mediated killing, expressed Snai1 and stem cell antigen-1 (Sca-1), and exhibited hybrid epithelial-mesenchymal features (114). SOX2, another transcription factor associated with EMT, was reported to promote resistance to ICBs (115). Additionally, Dongre et al. found that quasi-mesenchymal cells can not only resist anti-CTLA-4 therapy but also protect epithelial cells from immune attack (116). Tumor cell plasticity is a concept that easy to understand but hard to research. The high-resolution technologies may help the related research, such as single-cell RNA-seq (47) and spatial multi-omics (48).

### Tumor Extrinsic Mechanisms Underlying AR

The main tumor extrinsic mechanism for AR is the upregulation of other immune checkpoints, such as TIM-3, LAG-3, and VISTA (117). With tumor progression, other immune checkpoints are sequentially upregulated, with PD-1 upregulated as an early event, while LAG-3 and BTLA upregulated as a late event (118). BTLA has two classical inhibitory motifs, ITIM and ITSM, and an immune activating motif Grb2 (119, 120). Ritthipichai et al. reported that CD8^+^BTLA^+^ tumor- infiltrating lymphocytes exerted better anti-tumor immunity than the BTLA^-^ counterpart (120). Therefore, the specific contribution of BTLA to AR needs further exploration. Moreover, T cells with PD-1 high expression are more severely exhausted and show poorer response to PD-1 inhibitors, while T cells with PD-1 moderate expression can be saved by PD-1 inhibitors (118). Riaz et al. found that multiple immune checkpoints were upregulated during nivolumab treatment (41). Through mouse models with AR to PD-1 inhibitors, Koyama et al. found that PD-1 inhibitors were still bound to the T cells in drug-resistant specimens, but TIM-3 upregulation resulted in drug resistance (121). In addition, the anti-TIM-3 antibody was able to overcome the drug resistance, but CTLA-4 and LAG-3 were upregulated sequentially (121). Furthermore, they also reported two cases of AR to PD-1 inhibitors, in which TIM-3 was upregulated (121). Shayan et al. found that the PD-1 inhibitors induced TIM-3 upregulation was achieved through the PI3K-Akt pathway (122). Recently, the TIM-3/Galectin-9 pathway has been shown to induce primary resistance and AR to ICBs (123). In the preclinical study, Huang et al. found that when one of these three immune checkpoints, PD-1, LAG-3, and CTLA-4, was inhibited, the other two were upregulated, thus limiting the efficacy of the single inhibitor (124). In the cohort of NSCLC patients with AR to ICBs (13), eight patients were able to obtain matched samples before and after treatment, of which three patients were detected with TIM-3 upregulation, five with LAG-3 upregulation. In addition, the three patients with TIM-3 upregulation also demonstrated a co-upregulation with LAG-3 (13). MHC-II is a ligand of LAG-3 (125). For the MHC-II positive tumors, not only LAG-3 was upregulated in the resistant specimens, but also FCRL6, another receptor of MHC-II, was upregulated and suppressed the immune function (126). FCRL6 might be an emerging immune checkpoint (126). Furthermore, their group also found that the upregulation of TIM-3 and LAG-3 was not associated with primary resistance, but only with AR (126).

Gao et al. first reported the upregulation of VISTA in prostate cancer patients after CTLA-4 inhibitor treatment (127). In the cohort of melanoma patients with AR to ICBs (95), twelve patients were able to obtain matched samples before and after treatment, eight of which were detected with VISTA upregulation.

TIGIT was first identified by Yu et al. in 2009 as an immune checkpoint rheostat that suppresses the activation of T cells (128). TIGIT is mainly expressed on T cell subsets (including Tregs and memory T cells) and NK cells (128, 129). Indeed, a preclinical study showed that TIGIT blockade can prevent NK cell exhaustion and elicit potent anti-tumor immunity (130). A preliminary phase II clinical trial showed that the combination of TIGIT blockade and anti-PD-L1 antibody has better objective response rate (ORR) and median Progression Free Survival (mPFS) than anti-PD-L1 antibody alone in the first line setting (131). However, the specific contribution of TIGIT to AR is still not clear. It is unknown whether the superiority of the combination with TIGIT blockade can be translated into later line settings, which means conquering the real AR to prior immune checkpoints.

Although the upregulation of other immune checkpoints has been considered the main tumor extrinsic mechanism to AR, some other tumor extrinsic factors also potentially result in AR and need further research, including tumor vasculature, systemic and tumoral metabolic status, microbiome, and multiple cytokines.

## Potential strategies to Overcome AR

Understanding the clinical characteristics and underlying mechanisms of AR is ultimately required for overcoming them. As mentioned above, the mechanisms of AR are highly diversified, including neoantigen depletion, defects in antigen presentation machinery, aberrations of interferon signaling, tumor-induced exclusion/immunosuppression, tumor cell plasticity, and upregulation of other immune checkpoints. Patients may resist ICBs through one of these mechanisms or a combo of them. So, it is hard to find one strategy to fit all the situations. The more executable strategy is to apply a set of treatment modalities to patients with the same clinical characteristics or resistance mechanisms (**Table 3**).

**Table 3:** The potential strategies to overcome acquired resistance.

Clinical characteristics or resistance mechanisms	Potential strategies
Oligo-progression	Continuous ICB plus local therapy (132–136)
Neoantigen depletion	Oncolytic virotherapy such as T-VEC (137–141)
	Continuous ICB plus vaccine (142)
	Continuous ICB plus superantigens (143)
Defects in antigen presentation machinery	NK cell-based therapy (144)
	Continuous ICB plus RIG-I activation (145)
	Continuous ICB plus overexpression of NLRC5 or intratumoral delivery of BO-112 (146)
Aberrations of interferon signaling	T cell–based adoptive cell therapy (146)
Tumor-induced exclusion/immunosuppression	Continuous ICB plus inhibition of the involved pathways, including Wnt/β-catenin, PI3K-Akt, IFN-β/NOS2, CSF-1, TGF-β, adenosine, and IDO
	Continuous ICB plus microenvironment-targeting strategies (147)
Tumor cell plasticity	Continuous ICB plus epigenetic modulation (148, 149)
	Continuous ICB plus MRD-targeting strategies (150)
	Continuous ICB plus EMT inhibition
	Continuous ICB plus ferroptosis induction (151–153)
	Continuous ICB plus other plasticity-targeting strategies (107, 108)
Other immune checkpoints upregulation	Continuous ICB plus inhibition of the upregulating checkpoints
Other strategies	Continuous ICB plus chemotherapy, anti-angiogenesis therapy, radiotherapy, target therapy, or another immune checkpoint inhibitor
	Stop using ICB and to use later-line chemotherapy

### Strategies for Oligo-Progression

Since most patients with AR to ICBs develop oligo-progression (**Table 2**), local therapy becomes the most available way to overcome resistance. Radiotherapy can not only control the local disease but also shows a synergistic effect on immunotherapy (132, 133), thus being a preferred choice. Local resection is another choice worthy of consideration that has been proved safe for patients with advanced NSCLC after ICB therapy (134, 135). Cryotherapy also seems feasible under certain conditions (136). In the Keynote 006 study, three patients received local resection and a second-course pembrolizumab therapy after the single-site progression with initial complete response, two of whom achieved a second-course complete response while one achieved stable disease (26). In a retrospective cohort of 26 patients with NSCLC, 15 patients who received local therapy achieved better overall survival than the total population (27).

### Strategies for Neoantigen Depletion

Oncolytic virotherapy is an effective way to elicit new immunogenicity for tumors lacking in neoantigen (137–140). In the case reports of talimogene laherparepvec (T-VEC) treatment to overcome AR to ICBs in melanoma patients, three of six patients were evaluated as PR (141). Simultaneous anti-PD-1 and vaccine therapy can also reverse resistance (142). Furthermore, superantigens (SAgs) treatment emerges as a promising strategy to elicit immunogenicity (143). The detailed approaches to heat the cold tumor can be referred to in another review (20).

### Strategies for Defects in Antigen Presentation Machinery

As for the defects in antigen presentation machinery, the coping strategy is either repairing the defects or activating the anti-tumor immunity independent of antigen presentation. However, the current knowledge about both approaches is very lacking. NK cell-based therapy might be an effective way to overcome this type of resistance, for the MHC-I loss is an activating signal for NK cell toxicity (144). RIG-I activation is also a promising strategy (145). Kalbasi et al. reported that overexpression of NLRC5 (nucleotide-binding oligomerization domain-like receptor family caspase recruitment domain containing 5) and intratumoral delivery of BO-112, a potent nanoplexed version of polyinosinic: polycytidylic acid (poly I: C), each restored MHC-I expression (146).

### Strategies for Aberrations of Interferon Signaling

Defects in tumor-intrinsic interferon (IFN) signaling fail ICBs against cancer, but these tumors still maintain sensitivity to the T cell-based adoptive cell therapy (ACT). Kalbasi et al. reported that ACT was effective against tumors with JAK2 loss (146). As tumors with JAK1 loss show significant downregulation of MHC-I, ACT combined with overexpression of NLRC5 or intratumoral delivery of BO-112 was an effective approach (146). As for the IFN-β induced resistance, IFN-β inhibition might be an effective strategy.

### Strategies for Tumor-Induced Exclusion/Immunosuppression

Since tumor-induced exclusion/immunosuppression involves the activation of multiple signaling pathways, pharmacological inhibition would be effective in this regard, including pathways of Wnt/β-catenin, PI3K-Akt, IFN-β/NOS2, CSF-1, TGF-β, adenosine, and IDO. Additionally, microenvironment-targeting combinations are also the future directions of cancer immunotherapy and can be referred to in another review (147).

### Strategies for Tumor Cell Plasticity

Epigenetic modulation is an effective approach to restrict tumor cell plasticity, but concrete strategies need to be explored (148, 149). Targeting minimal residual disease (MRD) is worthy of consideration for decreasing the tumor load to restrict plasticity (150). Selective inhibition of the EMT program might also help overcome AR to ICBs. Tsoi et al. found that melanoma can be categorized into four subtypes following a differentiation trajectory with subtype-specific sensitivity to ferroptosis induction. This presents a therapeutic approach to target the differentiation plasticity to increase the efficacy of immunotherapy (151). Besides, induction of ferroptosis has other immune-activating functions (152, 153). The detailed strategies to target tumor cell plasticity can be referred to in the other reviews (107, 108).

### Strategies for Upregulation of Other Immune Checkpoints

Theoretically, the sequential inhibition of other immune checkpoint receptors such as TIM-3, LAG-3, and VISTA can help overcome AR. Indeed, dozens of early-phase clinical trials have been initiated to explore the clinical efficacy of these checkpoint blockades with or without another checkpoint blockade, which can be referred to in other reviews (154–156). TIGIT is expressed on both T cells and NK cells and TIGIT blockade can elicit NK cell-based anti-tumor immunity (128–130), which might have specific significance for patients with AR to T cell-based ICB therapy. Besides, a phase II clinical trial has showed that TIGIT blockade can synergize with anti-PD-L1 antibody (131). The TIGIT blockade combinations are also promising candidates to overcome AR. It should be noted that the line of the designed therapy is important with regard to overcoming AR. Even if a combination strategy shows superior efficacy in the first-line setting, whether it can overcome AR to prior immune checkpoint blockades (in the second-line setting after prior ICB failure) is still unanswered. Above all, the evidence from these clinical trials and real-world studies is awaited.

### Other Strategies

Since the guidelines or expert opinions about the treatment after ICB failure are scarce, there are various approaches in clinical practice to treat patients with AR to ICBs. Continuation of prior ICB combined with other therapies, such as chemotherapy, anti-angiogenesis therapy, radiotherapy, target therapy, and another immune checkpoint inhibitor, is frequently used. Another approach usually used is by changing immunotherapy into classical later-line modalities of chemotherapy. The evidence to support these attempts is at its infancy and arises a core question as to whether to stop using the prior ICBs. The INSIGNA study (https://clinicaltrials.gov, NCT03793179) will preliminarily answer the question about which is superior between prior ICB plus chemotherapy and chemotherapy alone for the patients with AR to ICBs.

## Discussion

With the increasing use of ICBs in clinical practice, a large number of patients develop AR. However, the knowledge about AR to ICBs is limited and poorly summarized. Through reviewing the published papers associated with this term, we clarified the principal elements of it. The definition of AR to ICBs is inconsistent at present, but expert opinions are available for this question. Oligo-progression is the main resistance pattern. The underlying mechanisms of AR involve tumor intrinsic factors and extrinsic factors. Neoantigen depletion can protect tumor cells from immune attack, however, it is unclear whether the depletion of neoantigens is a consequence of Darwinian natural selection with the neoantigen-expressing clones being eradicated or is a biological adaptation at an individual cancer cell level. Antigen presentation deficiency can also protect tumor cells from being recognized and killed by the immune cells, which includes biallelic *B2M* mutation and *HLA-I* down-regulation. IFN-γ is a crucial mediator of anti-tumor immunity. The defects in the IFNG pathway can lead to AR, while the prolonged IFN-γ exposure can also elicit resistance by upregulating IDO and other immune checkpoint ligands. Under the pressure of immune elimination, tumor cells can induce an immunosuppressive microenvironment through the genetic approaches (*Wnt/β-catenin* activation and *PTEN* loss) and the non-genetic approaches (recruiting Treg, TAM, and MDSC and upregulating IDO, adenosine, and TGF-β pathway). Tumor cell plasticity preserves the great potential for AR. One important issue is transdifferentiation from NSCLC cells to SCLC cells, the other is the EMT program. The tumor extrinsic mechanisms of AR are mainly through the upregulation of other immune checkpoints, such as TIM-3, LAG-3, and VISTA. The potential strategy to overcome AR is to apply a set of modalities to patients with the same clinical characteristics or resistance mechanisms. Although not discussed in this review, monitoring resistance is an important issue, and the circulating tumor DNA could be an effective tool.

One of the future directions in this field is to explore the mechanisms underlying AR to ICBs more comprehensively. New tools, such as CRISPER screen, single-cell RNA sequencing, and spatial multi-omics, will fuel the related research. It is worthy of noting that many mechanisms of primary resistance to ICBs have not been completely elucidated in the field of AR. For example, gut microbiota and tumor metabolism have been acknowledged to play important roles in primary resistance to ICBs (157–159), while their contribution to AR is largely unknown and thus needs future exploration. The other issue in this field is that translational research is urgently needed to extend patients’ survival. Through efforts to overcome AR, immunotherapy can be a promising therapeutic modality for curing cancer.

## Author Contributions

QC conceptualized this review. BZ searched the papers and drafted the manuscript. YG, PZ, and QC revised the manuscript. All authors contributed to the article and approved the submitted version.

## Funding

This work was supported by the Natural Science Foundation of China (Grant No. 82072597 and No. 81974483).

## Conflict of Interest

The authors declare that the research was conducted in the absence of any commercial or financial relationships that could be construed as a potential conflict of interest.
